# The Developmental Nature of the Victim-Offender Overlap

**DOI:** 10.1007/s40865-017-0068-3

**Published:** 2017-10-09

**Authors:** Amber L. Beckley, Avshalom Caspi, Louise Arseneault, J. C. Barnes, Helen L. Fisher, Honalee Harrington, Renate Houts, Nick Morgan, Candice L. Odgers, Jasmin Wertz, Terrie E. Moffitt

**Affiliations:** 1 Department of Psychology and Neuroscience, Duke University, Durham, NC USA; 2 Demography Unit, Stockholm University, Stockholm, Sweden; 3 Department of Psychiatry and Behavioral Sciences, Duke University, Durham, NC USA; 4 Centre for Genomic and Computational Biology, Duke University, Durham, NC USA; 5 MRC Social, Genetic & Developmental Psychiatry Centre, Institute of Psychiatry, Psychology & Neuroscience, King’s College London, London, UK; 6 School of Criminal Justice, University of Cincinnati, Cincinnati, OH USA; 7 Home Office Science, London, UK; 8 Center for Child and Family Policy and the Sanford School of Public Policy, Duke University, Durham, NC USA

**Keywords:** Victim-offender overlap, Developmental criminology, Adverse childhood experiences, Low selfcontrol

## Abstract

**Purpose:**

It is well-established that victims and offenders are often the same people, a phenomenon known as the victim-offender overlap, but the developmental nature of this overlap remains uncertain. In this study, we drew from a developmental theoretical framework to test effects of genetics, individual characteristics, and routine-activity-based risks. Drawing from developmental literature, we additionally tested the effect of an accumulation of adverse childhood experiences (ACEs).

**Methods:**

Data came from the Environmental Risk (E-Risk) Study, a representative UK birth cohort of 2232 twins born in 1994–1995 and followed to age 18 (with 93% retention). Crime victimization and offending were assessed through self-reports at age 18 (but findings replicated using crime records). We used the classical twin study method to decompose variance in the victim-offender overlap into genetic and environmental components. We used logistic regression to test the effects of childhood risk factors.

**Results:**

In contrast to past twin studies, we found that environment (as well as genes) contributed to the victim-offender overlap. Our logistic regression results showed that childhood low self-control and childhood antisocial behavior nearly doubled the odds of becoming a victim-offender, compared to a victim-only or an offender-only. Each additional ACE increased the odds of becoming a victim-offender, compared to a victim-only or an offender-only, by approximately 12%, pointing to the importance of cumulative childhood adversity.

**Conclusions:**

This study showed that the victim-offender overlap is, at least partially, developmental in nature and predictable from personal childhood characteristics and an accumulation of many adverse childhood experiences.

**Electronic supplementary material:**

The online version of this article (10.1007/s40865-017-0068-3) contains supplementary material, which is available to authorized users.

## Introduction

The connection between crime victimization and offending, known as the victim-offender overlap, is a well-established “fact” in criminology [63]. Victimization and offending both peak in adolescence and have been shown to be significantly associated with one another regardless of the type of data used, or the type of offending and victimization analyzed [66]. The significant overlap between victimization and offending appears across different countries and ethnic groups and applies to both violent and non-violent crime [62, 72, 92, 93, 101]. Studies report that upwards of half of victims are offenders and vice-versa [63] and the size of the correlation between victimization and offending is large, relative to other effect sizes found in criminology [61, 94, 95] (e.g., *r* = 0.30–0.50 [6]; *r* = 0.29 [39]; *r* = 0.24 [108]; *r* = 0.42 [114]). Victimization and offending are so intertwined that, some have argued, the understanding of one necessitates consideration of the other [66].

There has, however, been a modest amount of effort devoted to understanding the developmental etiology of the overlap despite a call for the investigation into biological mechanisms behind the overlap [66], and despite evidence that childhood risk factors, such as low self-control, are common risks for both adolescent victimization and offending [94, 95]. The lack of studies on the developmental nature of the overlap appears to be rooted in a lack of data. There are only two published studies on the genetic underpinnings of the victim-offender overlap [6, 118]. Additionally, we are not aware of any other published study that has examined the link between childhood risk factors, as assessed during the first decade of life, and later victim-offender overlap, and datasets for doing this work are few. Childhood risk factors have been identified in studies of offending, and some of the same childhood risk factors have been identified in separate studies of victimization, but few studies can test which risk factors differentiate victim-offenders from comparison groups of pure offenders and pure victims.

Our study adds to the body of research on the victim-offender overlap by addressing its developmental nature using the Environmental Risk (E-Risk) Study, a population-representative prospective longitudinal study of a 1994–1995 birth cohort of 1116 twin pairs from England and Wales, in which victimization and offending were assessed at age 18 years. Our twin design enabled us to add to the extant research by taking a behavioral genetic approach to the study of the victim-offender overlap. We additionally included prospective early- and middle-childhood risks measured up to age 12. Our study provides new evidence on the effect of cumulative adverse childhood experiences (as opposed to any individual experience) on the victim-offender overlap using both variable- and person-centered approaches. Our multimethod approach goes beyond past studies, which have typically relied on single measurement methods (e.g., self-report only) and a single analytical approach. We begin by briefly reviewing the literature on the victim-offender overlap. For a complete review of theory and past research on the overlap, we direct readers to three excellent reviews: Jennings et al. [63], Lauritsen and Laub [66], and Schreck and Stewart [111].

## Past Victim-Offender Overlap Research from a Developmental Perspective

Three general streams of research form the basis of knowledge on the victim-offender overlap. The first stream of research concerns the influence of genetics on the victim-offender overlap. Children may be genetically predisposed to develop certain characteristics (e.g., low self-control, substance use) which are related to both victimization and offending. Genetically influenced characteristics may also evoke an environment conducive to the victim-offender overlap. For example, genes influence poor educational attainment [11, 83], which may, in turn, create an environment *conducive* to becoming a victim-offender. To date, two twin studies have reported substantial genetic influence on the victim-offender overlap [6, 118]. In addition, research on bullying has pointed to the importance of genetic factors in explaining the overlap between bullying perpetration and bullying victimization in children [5]. Although behavioral genetic methods have been a source of controversy in criminology [8, 18, 77], these provocative findings merit further replication efforts.

A second stream of victim-offender overlap research focuses on individual heterogeneity. This research flags individual-level characteristics as the key to the victim-offender overlap [12, 13, 66]. In line with the general theory of crime [50], it has been argued that the victim-offender overlap is due to low self-control [111]. Many studies, however, have measured low self-control concurrently with victimization and offending, leaving open the possibility of reverse causation, in which being a victim-offender diminishes self-control.

The dual taxonomy of antisocial behavior [75] also identifies individual-level characteristics that may be linked to the victim-offender overlap. Low cognitive ability, shown to be distinct from low self-control [76], may impede the decision-making process where risks and rewards are evaluated, thereby increasing the risk of offending. Research has consistently linked low cognitive ability with offending [54] and has implied that low cognitive ability may also increase the risk of victimization [4, 10, 106, 107]. The dual taxonomy also identifies early puberty as a risk for the victim-offender overlap. Youths experiencing early puberty may have a prolonged maturity gap in which they attempt to mimic “adult” behaviors, which manifest as crime, but which also expose youths to the risk of victimization [75]. Young adolescents experiencing early puberty may be at increased risk of crime victimization through unstructured socialization with older-aged peers [58] and by facing new developmental pressures before completing age-appropriate developmental tasks [88, 115]. However, empirical support for early puberty as a risk for the overlap, like low cognitive ability, comes from separate studies on offending and victimization [20, 25, 32, 42, 51, 53, 57, 58, 109, 121].

The third stream of victim-offender overlap research is rooted in routine activity theory. Despite the explicit situational focus of routine activity theory [86], the constructs identified by the routine activity framework can also be integrated into a developmental perspective on crime. Routine activity theory states that crime results from the non-random convergence of likely offenders, suitable targets, and a lack of capable guardians [23, 48, 59]. The non-random convergence of these elements is based on the similar lifestyles of potential offenders/targets. The theory flags a number of constructs which have been shown to be relevant for victimization and offending separately and/or the victim-offender overlap. In this research, we focus on: delinquency [1, 69, 106, 122], substance use and substance using peers [37, 41, 49, 67, 112], low parental monitoring [15, 21, 85, 86], and neighborhood crime and victimization [24]. Indeed, research shows that adolescent delinquency and adolescent substance use arise from developmental processes beginning as early as the neonatal stage and share notable behavioral predictors during childhood [55]; [70]; [71]. Low parental monitoring and neighborhood crime and victimization are not only factors that create situations conducive to crime, but they are also developmental risk factors that shape the attitudes and future behavior of children [26, 33, 47, 87].

## Understanding the Developmental Nature of the Victim-Offender Overlap Through Cumulative ACEs

Adverse childhood experiences (ACEs) are potentially preventable childhood risk factors for poor adult health and social problems [40]. The original ACEs list, recommended by the U.S. Center for Disease Control and Prevention, includes physical abuse, sexual abuse, emotional abuse, emotional neglect, physical neglect, domestic violence exposure, criminality in the household, household substance abuse, mental illness in household, and parental separation or divorce [2]. The hypothesized association between ACEs and the victim-offender overlap is suggested by Widom’s research on abused and/or neglected children which has shown, albeit in separate studies, that maltreated children are likely to grow up to be both crime victims and offenders [73, 74, 104, 119, 120]. Other studies in criminology have also documented the relationship between ACEs and the risk of later victimization or later offending, although they have not explicitly tested the contribution of these to the victim-offender overlap, per se [9, 17, 30, 38, 56, 60, 65, 68, 113].

Criminological theories flag a number of additional experiences that may be relevant to explaining the victim-offender overlap and that can be added as adjuncts to the conventional ACEs. The idea of “expanding” ACEs was solidified by the Philadelphia ACEs Survey [28]. From routine activity theory and the results of the Philadelphia ACEs survey, we identified 10 additional adversities that could be found in the E-Risk Study dataset and that are potentially important to the victim-offender overlap: experiencing bullying, living in foster care, low childhood socioeconomic status, peer substance abuse, low parental monitoring (as evaluated by parents), low parental monitoring (as evaluated by children), participant-perceived unsafe neighborhood, high neighbor crime victimization measured via neighbor survey, neighborhood rated as unsafe through systematic social observation, and high-crime neighborhood measured through official police records.

In line with developmental theories of risk, the effect of ACEs is cumulative in nature [40]. Cumulative effects have not yet been considered in victim-offender overlap research.^1^ Indeed, past overlap research has controlled for many factors simultaneously and has generally found weak effects of presumed risk factors. Including many risk factors separately in a single statistical model yields estimates for each individual risk while controlling for the other risks, but does not account for the effect that many accumulated risks may have on the individual as does a summed count measure of risks. Counting risk factors cumulatively is a useful way to reduce measurement error, increase validity, and simultaneously account for correlated risk factors [35, 36]. Studies have shown that even when each individual risk experience has a weak or nil effect, the accumulation of multiple risk experiences has greater detrimental developmental effects, i.e., the sum is greater than its parts [35, 36].

There are a few ways to understand how an accumulation of ACEs may influence the victim-offender overlap. First, accumulated ACEs are likely to lead children to have low-quality, or insecure, attachment to their caregivers [19, 27]. Children subsequently model poor parent-child relationships in other interpersonal relationships throughout life [22]. There is evidence that children with positive perceptions of their family climate are at a lower risk of crime victimization [110] and that poor caregiver attachment can result in children who are inhibited and anxious or hostile and aggressive [14], potential risks for becoming a victim-offender. Second, an accumulation of many ACEs may teach children that violence perpetration and violent victimization are normal, expectable, and even acceptable. Third, when accumulated ACEs include multiple forms of childhood maltreatment/neglect, low self-control may ensue, and thereby increase the risk of adolescent victimization and offending [111].

Fourth, the accumulation of many ACEs in a life may lead to pathophysiologic consequences likely to increase the risk of offending [3, 29]. Exposure to chronic stress, in the form of multiple ACEs, while the brain is developing may lead to structural and functional abnormalities in regions of the brain (the prefrontal cortex, the amygdala, and the hippocampus) linked to crime and antisocial behavior [100]. Cortisol levels, also affected by chronic stress, may interact with testosterone to increase antisocial behavior [31, 46, 79]. Fifth, some ACEs are known to be under genetic influence (e.g., familial substance abuse or mental illness, family violence), which implies that the statistical prediction from ACEs to the later victim/offender overlap could, in part, operate through the aforementioned genetic pathways [91].

## The Present Study

In this study, we analyzed data on 18-year-old twin pairs to test explanations of the overlap between victimization and offending behavior. We first established that the overlap between victim status and offender status exists in the present cohort. We then exploited the twin design to test for genetic and environmental contributions to the victim-offender overlap. We further exploited the study’s prospective longitudinal design to test hypotheses that childhood risk factors would predict the victim-offender overlap. We drew from theories of individual heterogeneity and routine activity theory to test hypotheses that childhood personal risk factors predicted the victim-offender overlap. We additionally created a count reflecting each child’s accumulation of conventional ACEs and expanded ACEs, which included experiential factors (i.e., factors external to the child) flagged by routine activity theories, to test their ability to predict the victim-offender overlap.

Using variable-centered and person-centered approaches, we tested the hypotheses that

- H1: Both genes and environment contribute to the victim-offender overlap.
- H2: Childhood personal risk factors positively contribute to the victim-offender overlap.
- H3: The accumulation of adverse childhood experiences (ACEs) positively contributes to the victim-offender overlap.

## Data

### Participants

Participants were members of the Environmental Risk (E-Risk) Longitudinal Twin Study, which tracks the development of a birth cohort of 2232 British children. The sample was drawn from a larger birth register of twins born in England and Wales in 1994–1995 [117]. Full details about the sample are reported elsewhere [78]. Briefly, the E-Risk sample was constructed in 1999–2000, when 1116 families (93% of those eligible) with same-sex 5-year-old twins participated in home-visit assessments. Follow-up home visits were conducted when the children were aged 7 (98% participation), 10 (96% participation), 12 (96% participation), and, in 2012–2014, 18 years (93% participation). There were no differences between those who did and did not take part at age 18 in terms of socioeconomic status (SES) assessed when the cohort was initially defined (*χ* ^2^ = 0.86, *p* = .65), age 5 IQ scores (*t* = 0.98, *p* = .33), or age 5 internalizing or externalizing behavior problems (*t* = 0.40, *p* = .69 and *t* = 0.41, *p* = .68, respectively). The sample includes 55% monozygotic and 45% dizygotic twin pairs; sex is evenly distributed within zygosity (49% male). We have, in our analyses of the E-Risk data, adjusted the standard errors and confidence intervals for clustering of twins within families.

Families were recruited to represent the UK population of families with newborns in the 1990s, on the basis of residential location throughout England and Wales, and mother’s age. Teenaged mothers with twins were overselected to replace high-risk families who were selectively lost to the register through nonresponse. Older mothers having twins via assisted reproduction were underselected to avoid an excess of well-educated older mothers. These strategies ensured that the study sample represents the full range of socioeconomic conditions in Great Britain, as reflected in the families’ distribution on a neighborhood-level socioeconomic index (ACORN [A Classification of Residential Neighborhoods], developed by CACI Inc. for commercial use) [82]: 25.6% of E-Risk families live in “wealthy achiever” neighborhoods compared to 25.3% nationwide, 5.3 vs. 11.6% live in “urban prosperity” neighborhoods, 29.6 vs. 26.9% live in “comfortably off” neighborhoods, 13.4 vs. 13.9% live in “moderate means” neighborhoods, and 26.1 vs. 20.7% live in “hard-pressed” neighborhoods. E-Risk underrepresents “Urban Prosperity” because such households are significantly more likely to be childless. Since study participants live all over England and Wales, there was no geographic clustering (beyond twins within families) of individuals within neighborhood locations.

### Victimization at Age 18 Years

To assess victim status, participants were interviewed face-to-face at age 18 years about their crime victimization experiences between ages 12 and 18 years, which are the years in secondary school in the UK (a meaningful reporting period for the respondents). Face-to-face interviews were conducted to allow the clinical interviewer to administer probes and also to respond if a twin became distressed. Standardized interviews used the Juvenile Victimization Questionnaire 2nd revision (JVQ-R2) [44, 52], adapted as a clinical interview [45]. The JVQ-R2 was developed at the University of New Hampshire Crimes Against Children Research Center, has good psychometric properties [43], has been used extensively in nationwide US surveys (including the National Survey of Children’s Exposure to Violence), and was used in the UK National Society for the Prevention of Cruelty to Children (NSPCC) national survey [98, 99]. Study members were asked whether they had been a victim of a variety of types of physical assault (10 types), property crime (3 types), sexual assault (4 types), or internet/mobile phone harassment (3 types).^2^

Affirmative (“yes”) responses to each of the 20 victimization questions were summed to create a victimization variety score. Forty-eight percent of Study members reported at least one type of victimization and, on average, Study members reported 1.52 different types of victimization each. E-Risk Study members’ victimization was similar to that of lifetime victimization reported for the NSPCC sample [45]. See Tables S.1 and S.2 for descriptive statistics about victimization.

### Offending at Age 18 years

To assess offender status, participants completed a computer questionnaire at age 18 years which asked about their engagement in a variety of types of offending behaviors between ages 17 and 18 years. A computer questionnaire, as opposed to a face-to-face interview, was administered as distress was not expected and validity of reporting illegal behaviors by teens is enhanced by a computer format [103]. The respondent was able to read the short description of each crime on the screen, while also hearing it over headphones to eliminate any effects of poor reading skill. There were 20 questions about non-violent offenses such as theft, fraud, vandalism, breaking and entering, and selling drugs. There were 13 questions about violent offenses such as assault, robbery, making threats, and carrying a weapon.

Affirmative (“yes”) responses to each of the 33 offending questions were summed to create an offending variety score. Fifty percent of Study members reported any type of offending and, on average, Study members reported 1.74 different types of offending each. See Table S.1 for descriptive statistics about offending.

### Personal Risk Factors Ages 5–12 Years

We included the following childhood personal risk factors that have been theoretically linked to the victim-offender overlap: low self-control, low cognitive ability, early puberty, conduct disorder diagnosis, pre-teen delinquent behavior, and pre-teen substance use. These risk factors were measured during childhood up to age 12 years, preceding the measurement of both victimization and offending, providing stronger grounds for causal inference based on temporal order. Measurement details are provided in Table S.1.

### Accumulation of Adverse Childhood Experiences

We measured ACEs in two categories. First, we measured conventional ACEs [40]: physical abuse, sexual abuse, emotional abuse and neglect, physical neglect, domestic violence exposure, parental antisocial behavior, family history of substance abuse, family history of mental health disorders, and parental separation or divorce. Second, from the results from the PHL ACEs survey and routine activity theory, we identified 10 adversities that we could test as part of the E-Risk Study: experiencing bullying, living in foster care, low childhood socioeconomic status, peer substance abuse, low parental monitoring (as evaluated by parents), low parental monitoring (as evaluated by children), participant-perceived unsafe neighborhood, high neighbor crime victimization measured via neighbor survey, neighborhood rated as unsafe through systematic social observation, and high-crime neighborhood measured through official police records. Sixteen ACEs were measured during childhood up to age 12 years; 3 neighborhood ACEs were measured between ages 13 and 17; measurement details are provided in Table S.1. The correlations between the items in our ACEs scale can be found in Table S.3.

We additionally created a summary scale of all risk factors. The summary scale of all risk factors added conventional ACEs, expanded ACEs, and personal risk factors. Study members were counted as having the personal risk factor if they were in the top quartile of low self-control, early puberty, childhood self-reported delinquency, and childhood substance use; in the bottom quartile of cognitive ability; met the diagnostic criteria for conduct disorder.

All analyses were carried out in R 3.3.1 [97] using the following packages: OpenMx [16, 80, 96], ppcor [64], and compareGroups [116].

## Results

Our preliminary analytic step was to verify that the victim-offender overlap existed in our data. Study members were about 1.5 times as likely to be victim-offenders (29%) compared to victims-only (16%) or offenders-only (20%). We assessed the association between self-reported victimization and offending through a correlation. E-Risk data confirmed the association between victimization and offending. Adolescents who were victimized were significantly more likely to offend (*r* = 0.42, 95% CI 0.39–0.46).

### Do Both Genes and Environment Contribute to the Victim-Offender Overlap?

The first part of our main analysis was a behavioral genetic analysis to test the hypothesis that both genetic and environmental factors contribute to the victim-offender overlap. It was important to begin with a behavioral genetic model because (a) past research exploring environmental risk factors has produced few conclusive results explaining the victim-offender overlap and (b) past research using behavioral genetic models has found that environmental factors do not explain the victim-offender overlap [6, 118]. We used the classical twin study method to decompose variation in victimization, offending, and their overlap into three latent factors: additive genes, the “shared” environment, and the “unique” environment.^3^ The shared environment represents environmental non-genetic factors that make family members similar, whereas the unique environment represents factors that make family members different (and also includes measurement error in the outcome measure). The proportion of variance in a phenotype attributed to the three latent factors is estimated by comparing the degree of similarity between identical (monozygotic (MZ)) twins, who share 100% of their genetic variation, to the degree of similarity between fraternal (dizygotic (DZ)) twins, who share 50% of their genetic variation, on average. One key assumption of the twin study method is that monozygotic and dizygotic twin pairs experience trait-relevant environmental exposure equally (the equal environment assumption).^4^ In other words, the zygosity of the twin pair is not tied to environmental experiences; in practice, this biases result towards genetic influence. To estimate the relative importance of the genetic and environmental influences, we compare the correlation of a characteristic between Twin A and Twin B in pairs of MZ twins to that of the correlation of a characteristic between Twin A and Twin B in pairs of DZ twins. If twin correlations are greater for MZ pairs than for DZ pairs, this indicates genetic influences on individual differences in a characteristic. If the correlation between twins is equal across MZ and DZ pairs, this indicates shared environmental influences. If the correlation between MZ pairs is less than perfect, then we can infer that influences uniquely experienced by each child have made the twins different. We first estimated the twin correlations of victimization, offending, and their overlap. We then fit a bivariate model with Cholesky decomposition, which decomposes the variance of victimization and offending and their covariance into genetic, shared, and non-shared environmental influences. The best fitting model retained additive genetic, shared environmental, and unique environmental influences (model fit statistics can be found in Table S.4).

**Fig. 1:**
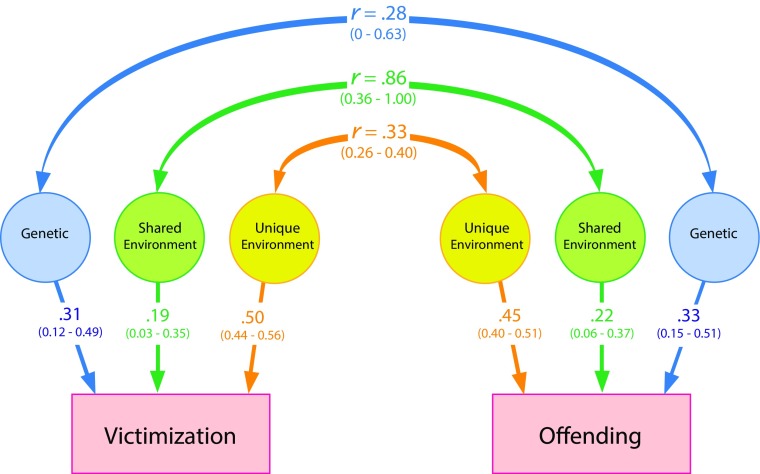
Correlated factor solution from the bivariate twin model. Univariate estimates showed that victimization and offending were influenced by genetic and environmental factors. Correlations showed that victimization and offending were affected by many of the same shared environmental factors

The correlation between Twin A’s victimization and Twin B’s victimization was significantly greater for MZ than for DZ pairs (0.51, 95% CI 0.44–0.57, in MZ vs 0.34, 95% CI 0.26–0.42, in DZ) (see Table 1). As implied by these correlations, modeling showed that the variation in victimization was explained by genetic factors (31%, 95% CI 0.12–0.49), shared environmental factors (19%, 95% CI 0.03–0.35), and unique environmental factors (50%, 95% CI 0.44–0.56) (see Fig. 1).^5^

**Table 1:** Estimated correlations between victimization and offending at age 18 years within and between twin pairs, by zygosity, 95% confidence interval in parentheses

	Victimization Twin B	Offending Twin B
Monozygotic		
Victimization Twin A	0.51 (0.44–0.57)	0.25 (0.17–0.32)
Offending Twin A	0.26 (0.18–0.34)	0.55 (0.49–0.60)
Dizygotic		
Victimization Twin A	0.34 (0.26–0.42)	0.24 (0.15–0.33)
Offending Twin A	0.22 (0.13–0.31)	0.39 (0.31–0.46)

The correlation between victimization and offending in the full cohort was 0.42. The correlation among monozygotic twins (0.42; 95%CI 0.35–0.48) and dizygotic twins (0.44; 95%CI 0.35–0.51) was not significantly different from the full cohort. The cross-twin cross-trait correlations were constrained to offending Twin A victimization Twin B in the bivariate Cholesky decomposition twin model

The correlation between Twin A’s offending and Twin B’s offending was also significantly greater for MZ than for DZ pairs (0.55, 95% CI 0.49–0.60, in MZ vs 0.39, 95% CI 0.31–0.46, in DZ). As implied by the correlations, modeling showed that the variation in offending was explained by genetic factors (33%, 95% CI 0.15–0.51), shared environmental factors (22%, 95% CI 0.06–0.37), and unique environmental factors (45%, 95% CI 0.40–0.51).

In contrast, the correlation between Twin A’s victimization and Twin B’s offending, and vice-versa (also known as the cross-twin, cross-trait correlation), was not significantly different between MZ and DZ twins (0.26, 95% CI 0.18–0.34, in MZ vs 0.22, 95% CI 0.13–0.31, in DZ; 0.25, 95% CI 0.17–032, in MZ vs 0.23, 95% CI 0.15–0.33, in DZ, respectively). This result implied that shared environmental factors were important for explaining the victim-offender overlap.

Given the result that both genes and environment were important for explaining victimization and offending, we next tested the extent to which the genetic and environmental factors influencing each outcome were similar by transforming the results of the Cholesky decomposition into the mathematically identical correlated factors solution (Loehlin 1996).^6^

The correlation (*r*) between the factors was estimated using the following formula;

$$ r=\frac{(vo)(v)}{\sqrt{\left({v}^2\right)\left({(vo)}^2+\left({o}^2\right)\right)}}, $$

where for a given factor (additive genetic, shared environment, unique environment), *vo* represents the unstandardized variance for victimization-offending, *v* represents the unstandardized variance for victimization, and *o* represents the unstandardized variance for offending.

Figure 1 shows that the genetic factors between victimization and offending were correlated at 0.28 (95% CI 0–0.63), indicating that victimization and offending had relatively little genetic influence in common. However, the shared environmental factors that accounted for the overlap between victimization and offending were correlated at 0.86 (95% CI 0.36–1.00), indicating that victimization and offending were influenced by many of the same shared environmental risk factors. The unique environmental factors between victimization and offending were correlated at 0.33 (95% CI 0.26–0.40), indicating that victimization and offending were influenced by some of the same unique environmental risk factors. Through path tracing, we calculated that the proportion of the covariance between victimization and offending that was explained by genetic factors was 22% (95% CI 0–0.56), by shared environmental factors 40% (95% CI 0.10–0.68) and by unique environmental factors 37% (95% CI 0.28–0.48). In sum, our results show that in this cohort, victimization and offending were under genetic and environmental influence. A large part of the overlap in victimization and offending could be attributed to many of the same (unmeasured) environmental factors.

We analyzed sex heterogeneity in the victim-offender overlap and found that, while there were some minor sex differences, the results between males and females were substantively similar. With about 500 pairs of boys and 500 pairs of girls, our cohort was not large enough to yield decisive tests of sex differences [102]. Sex-specific correlations followed the same pattern and can be found in Table S.6.

### Using a *Variable-Ventered* Approach, Do Childhood Personal Risk Factors and Adverse Childhood Experiences Positively Contribute to the Victim-Offender Overlap?

The second part of our analysis used a variable-centered approach to test the hypotheses that early childhood personal risk factors and the accumulation of adverse childhood experiences positively contribute to the victim-offender overlap. As in previous research, we found that the same risk factors that were associated with victimization were also generally associated with offending (see Table 2). Next, we tested the extent to which these risk factors accounted for the zero-order correlation between victimization and offending (which was *r* = 0.42, 95% CI 0.39–0.46). In this approach, we examined partial correlations between victimization and offending while controlling for each risk factor separately. We then calculated the amount of reduction in the correlation between victimization and offending, after partialling out common risk factors (see final column of Table 2). A reduced correlation would imply that the risk factor helps explain the victim-offender overlap.

**Table 2:** Association between risk factors, victimization (*v*) and offending (*o*) in a birth cohort. The table shows correlations between each risk factor with victimization and offending, respectively, and the partial correlation between victimization and offending, after partialling out effect of each risk factor

Risk factor	Correlation (*r*)		Partial correlation	
	*v*	*o*	*v-o* (*r*)	Reduces *v*-*o* zero-order correlation by (%)^a^
Personal risk factors				
Low self-control *z*-score	0.266	0.345	0.365	13.6
Cognitive ability	− 0.088	− 0.102	0.419	0.7
Early puberty	0.072	0.019^ns^	0.416	1.4
Conduct disorder diagnosis	0.208	0.320	0.384	9.1
Childhood self-reported delinquency	0.235	0.441	0.366	13.3
Childhood substance use	0.192	0.332	0.388	8.0
Total count of all 19 ACEs	0.274	0.257	0.378	10.4
Conventional ACEs (up to 9 ACEs)^b^	0.228	0.199	0.394	6.5
Physical abuse	0.187	0.140	0.407	3.6
Sexual abuse	0.052	0.061	0.420	0.4
Emotional abuse or neglect	0.143	0.089	0.415	1.6
Physical neglect	0.090	0.062	0.419	0.7
Domestic violence exposure	0.124	0.132	0.412	2.3
Parental antisocial behavior	0.161	0.151	0.405	3.9
Family history of substance abuse	0.148	0.104	0.416	1.4
Family history of mental health disorders	0.108	0.072	0.411	2.6
Parental separation or divorce	0.130	0.136	0.413	2.2
Expanded ACEs (up to 10 ACEs)	0.226	0.227	0.390	7.4
Bullying victim	0.147	0.023^ns^	0.423	−0.2
Lived in foster care	0.089	0.068	0.418	0.8
Low socioeconomic status	0.116	0.107	0.415	1.7
Peer substance abuse	0.168	0.242	0.400	5.2
Low parental monitoring (mother)	0.093	0.135	0.417	1.2
Low parental monitoring (participant)	0.107	0.213	0.412	2.4
Participant-perceived unsafe neighborhood	0.165	0.036^ns^	0.424	−0.6
High neighbor victimization	0.073	0.053	0.425	−0.7
Neighborhood rated unsafe	0.089	0.112	0.415	1.6
High-crime neighborhood	0.053	0.026^ns^	0.416	1.3
Summary scale of all risk factors^c^	0.232	0.361	0.372	11.7

Pairwise correlations for full sample

^a^Percent difference between zero-order correlation (0.42) and partial correlation

^b^Our measure includes 9 items, in contrast to the 10 conventional ACEs, because we found that coders could not empirically separate emotional abuse and emotional neglect in a reliable way

^c^The summary scale of all risk factors added conventional ACEs, expanded ACEs, and personal risk factors. Study members were counted as having the personal risk factor if they were in the top quartile of low self-control, early puberty, childhood self-reported delinquency, and childhood substance use; in the bottom quartile of cognitive ability; met the diagnostic criteria for conduct disorder

^ns^ *p* > .05. All other correlations significant *p* < .05, significance adjusted for within-family clustering

Table 2 shows that the risk factors, both personal risks and adverse childhood experiences, produced relatively weak reductions in the victimization-offending zero-order correlation. The strongest reductions came from the personal risk factors of low self-control and childhood self-reported delinquency, each of which reduced the zero-order correlation by 13%. The cumulative scale of all ACEs reduced the zero-order correlation by 11%. The summary scale of all personal risk factors combined with ACEs reduced the zero-order correlation by 12%, suggesting that personal risk factors and ACEs confer overlapping risk to the victim-offender overlap. As anticipated, when taken one-by-one, the single adversities contained in the ACEs scale produced weak reductions in the correlation between victimization and offending. The remaining variables (personal risk factors and the individual ACEs) all reduced the zero-order correlation between victimization and offending by less than 10% each.

### Using a *Person-Centered* Approach, Do Childhood Personal Risk Factors and Adverse Childhood Experiences Positively Contribute to the Victim-Offender Overlap?

In a complementary, person-centered approach, we categorized individuals into four groups: neither victims nor offenders (individuals scoring 0 on both variety score scales; hereafter “neither”, 35% of the sample), victims-only (individuals scoring 0 on offending variety but > 0 on victimization variety; 16%), offenders-only (individuals scoring 0 on victimization variety but > 0 on offending variety; 20%), or victim-offenders (individuals scoring greater than 0 on both victimization and offending; 29%). We tested whether victim-offenders differed on risk predictors from offenders-only, victims-only, or adolescents who were neither. It was not surprising that victim-offenders were consistently worse-off from those adolescents who were neither (Table 3). The interesting question was whether victim-offenders differed from victims-only and offenders-only. In terms of personal risk factors measured in childhood, victim-offenders, compared to all other groups, had lower self-control, were significantly more likely to be diagnosed with conduct disorder, self-reported delinquency and had experimented with substance use. In terms of adverse childhood experiences, victim-offenders, compared to both victims-only and offenders-only, had scored significantly higher on cumulative ACEs. Victim-offenders tended to be worse-off using the individual items from the ACEs scales, but this difference failed to reach statistical significance for many individual ACEs.

**Table 3:** Are victim-offenders unique? Comparing victim-offenders to three groups (individuals who are neither victims nor offenders, victims-only, and offenders-only)

	Victim-offender status				*p* < .05 compared to victim-offender^a^
	Neither (N) *n* = 659	Victim-only (V) *n* = 312	Offender-only (O) *n* = 380	Victim-offender *n* = 546	
Victimization and offending					
Victimization, mean (SD)	0.00 (0.00)	2.39 (2.09)	0.00 (0.00)	3.85 (2.91)	N, V, O
Offending, mean (SD)	0.00 (0.00)	0.00 (0.00)	2.64 (2.36)	4.07 (3.03)	N, V, O
Personal risk factors					
Low self-control z-score, mean (SD)	−0.37 (0.88)	−0.10 (0.89)	0.05 (0.99)	0.36 (1.02)	N, V, O
Cognitive ability, mean (SD)	99.9 (15.4)	98.4 (14.8)	100.2 (15.6)	98.1 (15.5)	–
Early puberty, mean (SD)	2.60 (1.08)	2.73 (1.13)	2.59 (1.11)	2.70 (1.12)	–
Conduct disorder diagnosis, *N* (%)	35 (5.3%)	35 (11.2%)	65 (17.1%)	151 (27.7%)	N, V, O
Childhood self-reported delinquency, mean (SD)	1.14 (1.39)	1.46 (1.52)	2.24 (2.25)	2.84 (2.11)	N, V, O
Childhood substance use, mean (SD)	0.54 (0.89)	0.70 (0.98)	0.88 (1.13)	1.30 (1.43)	N, V, O
Total of all ACEs (up to 19 ACEs), mean (SD)	2.30 (2.18)	3.31 (2.53)	2.97 (2.34)	4.15 (2.87)	N, V, O
Conventional ACEs (up to 9 ACEs), mean (SD)	1.09 (1.28)	1.66 (1.56)	1.34 (1.37)	2.00 (1.69)	N, V, O
Physical abuse, *N* (%)	17 (2.6%)	11 (3.5%)	11 (2.9%)	52 (9.5%)	N, V, O
Sexual abuse, *N* (%)	2 (0.3%)	2 (0.6%)	3 (0.8%)	9 (1.6%)	–
Emotional abuse or neglect, *N* (%)	6 (0.9%)	11 (3.5%)	8 (2.1%)	24 (4.4%)	N
Physical neglect, *N* (%)	3 (0.5%)	7 (2.2%)	2 (0.5%)	18 (3.3%)	N, O
Domestic violence exposure, *N* (%)	80 (12.1%)	54 (17.3%)	60 (15.8%)	127 (23.3%)	N, O
Parental antisocial behavior, *N* (%)	103 (15.6%)	95 (30.4%)	85 (22.5%)	183 (33.6%)	N, O
Family history of substance abuse, *N* (%)	94 (14.3%)	77 (24.7%)	73 (19.2%)	163 (30.0%)	N, O
Family history of mental health disorders, *N* (%)	167 (25.4%)	99 (32.0%)	96 (25.3%)	204 (38.2%)	N, O
Parental separation or divorce, *N* (%)	245 (38.0%)	163 (52.8%)	173 (46.0%)	310 (58.1%)	N, O
Expanded ACEs (up to 10 ACEs)	1.21 (1.38)	1.65 (1.51)	1.63 (1.51)	2.16 (1.84)	N, V, O
Bullying victim, *N* (%)	34 (5.2%)	41 (13.1%)	23 (6.1%)	65 (11.9%)	N, O
Lived in foster care, *N* (%)	2 (0.3%)	3 (1.0%)	1 (0.3%)	9 (1.6%)	–
Low socioeconomic status, *N* (%)	94 (14.3%)	56 (17.9%)	67 (17.6%)	135 (24.7%)	N, O
Peer substance abuse, *N* (%)	70 (10.6%)	53 (17.0%)	82 (21.7%)	169 (31.1%)	N, V, O
Low parental monitoring (mother), *N* (%)	86 (13.1%)	52 (16.7%)	80 (21.1%)	125 (22.9%)	N
Low parental monitoring (participant), *N* (%)	96 (14.6%)	52 (16.7%)	89 (23.4%)	165 (30.2%)	N, V, O
Participant-perceived unsafe neighborhood, *N* (%)	50 (7.6%)	43 (13.9%)	40 (10.6%)	99 (18.2%)	N, O
High neighbor victimization, *N* (%)	131 (20.5%)	90 (29.7%)	86 (23.4%)	156 (30.1%)	N
Neighborhood rated unsafe, *N* (%)	85 (14.4%)	56 (19.4%)	67 (19.4%)	124 (25.9%)	N
High-crime neighborhood, *N* (%)	148 (23.5%)	69 (22.8%)	84 (22.7%)	131 (25.4%)	–
Summary scale of all risk factors	1.57 (1.14)	1.95 (1.23)	2.14 (1.35)	2.66 (1.45)	N, V, O

*SD* standard deviation, *N* number of Study members

^a^Significant pairwise two-tailed tests between the victim-offender group and each of the other groups are noted by the single letter abbreviation of the group for which the significant difference was found: N = neither victim nor offender, V = victim-only, and O = offender-only. Mean differences tested with Tukey’s test. Proportion differences tested with chi-square or Fisher’s exact test with *p* values adjusted for multiple comparisons within rows using the method described by Benjamini and Hochberg (1995)

Next, we extended the analyses reported in Table 3 by controlling for (a) the variety of victimization and (b) the variety of offending. These controls were important because it is possible that any observed differences between victim-offenders and victims-only and offenders-only could arise simply because victim-offenders are more frequently victimized or are more frequent, high-volume offenders. For each of our eight key risk factors, we used logistic regression to test the likelihood of being a victim-offender versus being (a) a victim-only and (b) an offender-only, controlling for victimization frequency and offending frequency.

Table 4 reports the results of these logistic regression models in odds ratios with 95% confidence intervals. After controlling for adolescent victimization frequency, the odds that a Study member was identified as a victim-offender compared to a victim-only were still significantly increased by lower self-control (1.32, 95% CI 1.12–1.55), in the presence of a conduct disorder diagnosis (2.18, 95% CI 1.41–3.39), when more childhood delinquency was self-reported (1.45, 95% CI 1.31–1.60), when more childhood substance use was self-reported (1.48, 95% CI 1.26–1.73), when more ACEs were experienced (1.08, 95% CI 1.02–1.16), and with higher scores on the summary scale of all risks (1.35, 95% CI 1.19–1.52). After controlling for adolescent offending frequency, the odds that a Study member was identified as a victim-offender compared to an offender-only were still significantly increased by lower self-control (1.23, 95% CI 1.07–1.42), in the presence of a conduct disorder diagnosis (1.44, 95% CI 1.02–2.05), when more childhood substance use was self-reported (1.18, 95% CI 1.04–1.34), when more ACEs were experienced (1.15, 95% CI 1.09–1.22), and with higher scores on the summary scale of all risks (1.18, 95% CI 1.06–1.31).

**Table 4:** Association between personal risk factors and ACEs and being a victim-offender versus (a) a victim-only and (b) an offender-only

Risk factor	Victim-offender versus							
	Victim-only			Offender-only				
	Model 1 (covariate: male)		Model 2 (covariates: male, victimization)		Model 1 (covariate: male)		Model 2 (covariates: male, offending)	
	OR	95% CI	OR	95% CI	OR	95% CI	OR	95% CI
Personal risk factors								
Low self-control *z*-score	1.43	(1.22–1.69)	1.32	(1.12–1.55)	1.21	(1.11–1.32)	1.23	(1.07–1.42)
Cognitive ability	1.00	(0.99–1.01)	1.00	(0.99–1.01)	0.99	(0.99–1.00)	1.00	(0.99–1.01)
Early puberty	1.02	(0.89–1.17)	0.98	(0.85–1.13)	1.06	(0.98–1.15)	1.08	(0.95–1.22)
Conduct disorder diagnosis	2.47	(1.61–3.77)	2.18	(1.41–3.39)	1.45	(1.18–1.77)	1.44	(1.02–2.05)
Childhood self-reported delinquency	1.49	(1.35–1.65)	1.45	(1.31–1.60)	1.08	(1.03–1.13)	1.06	(0.98–1.14)
Childhood substance use	1.53	(1.31–1.79)	1.48	(1.26–1.73)	1.17	(1.08–1.27)	1.18	(1.04–1.34)
Total of all ACEs (up to 19 ACEs)	1.12	(1.05–1.19)	1.08	(1.02–1.16)	1.11	(1.07–1.15)	1.15	(1.09–1.22)
Summary scale of all risk factors	1.41	(1.25–1.59)	1.35	(1.19–1.52)	1.17	(1.10–1.24)	1.18	(1.06–1.31)

Victim-offender versus victim-only models *n* = 858. Victim-offender versus offender-only models *n* = 926. Models of model 1 type include the risk factor and male. Models of model 2 type include the risk factor, male, and victimization or offending variety

*OR* odds ratio, *CI* confidence interval

In summary, these models showed that in this cohort the victim-offender overlap could be partly explained through both personal risk factors and cumulative ACEs. We additionally conducted a sensitivity analysis using offending as recorded in the UK Police National Computer. Police records covered the period beginning at age 10 years, overlapping with the period covered by the self-reported victimization measure (details can be found in Table S.7). Consistent with our results using self-reports, low self-control, conduct disorder diagnosis, childhood self-reported delinquency, substance use, the scale of 19 ACEs, and the summary scale of all risk factors were all significantly more likely/higher among victim-offenders compared to victims-only. However, when using official records as the indicator of offending, offenders-only and victim-offenders did not appear significantly different from each other, probably because police-recorded offenders—whether victims or not—are in the upper end of the spectrum of risk.

## Discussion

Criminal justice policies have historically tended to contrast victims with offenders, but they are often the same people [66]. Our findings provide new evidence that during the peak age-period of crime victimization and offending, many victims are offenders and many offenders are victims. Moreover, victim-offenders seem to be characterized by early-onset antisocial behavior and are exposed to multiple adverse childhood experiences. Our findings make three novel contributions. First, our quantitative twin models showed that the victim-offender overlap is influenced by the environment and that victimization and offending have many environmental risks in common. Recall, prior studies had shown that most of the variation in the victim-offender overlap was due to genetic factors.

Second, in line with past developmental research comparing single risks to an accumulation of risks for other, non-crime, outcomes, our findings showed that childhood risk for the victim-offender overlap is cumulative in nature. Non-offending or not being victimized was associated with few or no risks. Being both a victim and an offender was associated with the largest accumulation of risks. Being either a pure victim or a pure offender fell in-between and was associated with a modest number of risks. Prior studies of the victim-offender overlap often reported that risk factors had little or no effect on the overlap, but this may have arisen from testing one risk factor at a time.

Third, we studied risk factors prospectively assessed during childhood, and our results illustrate that a developmental approach is useful for predicting the victim-offender overlap. Victim-offender overlap research can be further advanced through tests of other life course theories (e.g., the age-graded theory of crime) and through the use of longitudinal methods. This focus does not detract from the value and importance of research on the situational determinants of the victim-offender overlap. Many of the factors that we analyzed could easily predispose victims and offenders to switch roles during a single incident, or within a few days or weeks. This research aims to highlight the relevance of a longitudinal view of the overlap and highlight its developmental etiology. Current theories of crime over the life course should be augmented to consider victimization as an outcome predicted by similar risks as offending and an outcome likely to occur simultaneously with offending in the presence of an abundance of risk.

In line with meta-analyses on the heritability of antisocial behavior [102], our twin models supported our first hypothesis that both genes and environmental factors would be important for explaining the victim-offender overlap. Past research, in contrast, found that shared environmental factors had no significant effect on the victim-offender overlap [6, 118]. Sample differences in age could not explain the contrasting results, as the age range in our study was not noticeably different from ages covered by past research.^7^ One possible reason that our findings differed from those of past results is that the E-Risk Study has retained over 90% of its participants. In contrast, the Add Health Study retained about 75% of respondents between Waves I and II/III; non-participation and attrition conspire to reduce representation of people living in adverse homes. Additionally, the measure of victimization used in Add Health was limited to a few types of severe violent crime victimization, resulting in quite low prevalence and frequency of victimization—less than 25% of the sample had been victimized and the mean number of victimizations was less than 1. In contrast, our measure of victimization captured a wider variety of types of victimization, a few of which were family-based. The prevalence and frequency of victimization in our study are congruent with nationwide estimates of victimization derived from the NSPCC survey [45]. Regardless of particular data-related issues, a past meta-analysis of antisocial behavior [102] implies that many more behavioral genetic studies will be needed before drawing firm conclusions about genetic and environmental effects on the victim-offender overlap. Speaking to the broader controversy of using a twin design [8, 18, 77], our results showed that twin designs in criminology are valuable for showing environmental effects.

Our person- and variable-centered models partially supported our second hypothesis that personal risk factors would be important for explaining the victim-offender overlap. In the variable-centered approach, low self-control, conduct disorder diagnosis, childhood self-reported delinquency, and childhood substance use each explained a portion (≈ 8% or more) of the correlation between victimization and offending. Similarly, in the person-centered approach, Study members who had been diagnosed with conduct disorder, and those with high amounts of low self-control, childhood delinquency, and childhood substance use were significantly more likely to become a victim-offender versus a victim-only or an offender-only during adolescence. Our results thus supported the prediction from theories of individual heterogeneity that low self-control partly explains the victim-offender overlap. The two other personal risk factors, low cognitive ability and early-onset puberty, were not supported as risks for the victim-offender overlap. To the best of our knowledge, this is the first analysis to test the contribution of these two risk factors to the victim-offender overlap and replication of this novel null finding is needed.

Both our person- and variable-centered models supported our third hypothesis that cumulative adverse childhood experiences (ACEs) would be important for explaining the victim-offender overlap. The results of the variable-centered and person-centered approaches showed that individual ACEs, considered one-by-one, were unhelpful in explaining the victim-offender overlap. In contrast, cumulative ACEs produced relatively stronger reductions in the correlation between victimization and offending, differed significantly between victim-offenders and both victims-only and offenders-only, and significantly increased the odds of being a victim-offender versus an offender-only. Cumulative risk appeared especially important for neighborhood measures which, when measured individually, had little association with the victim-offender overlap.

## Limitations

There are limitations to our study. First, Study members were asked about their victimization over a six-year period (age 13–18), while they were asked about their offending over a one-year period (age 17–18). For our study, this meant that the overlap may have been based on events a number of years apart. A measure using the same 1-year exposure window may have led to fewer Study members reporting victimization and possibly fewer victim-offenders. Although the victim-offender overlap is often measured within a short period of time, many studies on the overlap between victimization and offending consider the offense history of crime victims (as did, for example, Wolfgang’s [123] landmark study, which was the first study to empirically establish the victim-offender overlap). In this regard, for our analysis, a longer window of offending may have been more appropriate than restricting the window of victimization. Recall that we were able to capture a multi-year window of offending (from age 10 onwards) with the police-reported offense data (Table S.7), and results were generally consistent with regard to the impact of the risk factors.

Second, we are limited in our ability to make causal inferences. We could not assess the temporal order of victimization and offending, only the overlap. We were, thus, unable to evaluate whether victimization caused offending or vice-versa. Additionally, our research can only support personal risk factors and cumulative adversities as indicators of risk for being a victim-offender and not necessarily as indicators of causation. Yet, randomized controlled experiments of parenting programs have connected lower childhood antisocial behavior and lower childhood maltreatment to lower adolescent delinquency and victimization [34, 84, 89, 124]. Thus, it would seem that reducing childhood antisocial behavior and ACEs could also lower the risk of children becoming adolescent victim-offenders. Moreover, given the nature of ACEs and the behavioral genetic results showing the influence of the shared environment, the family appears to be a good target for intervention.

Third, given the complexity of the current study, we have not delved further into how childhood risks may predict different types of victimization and offending. However, offense specialization in adolescence is uncommon [90] and many of our Study members were victims of more than one type of crime (see Table S.2). Future research should nonetheless consider the possibility of different childhood risks predicting different types of victimization and offending.

Fourth, the sample was composed of twins, so the results may not generalize to singletons. However, past studies of singletons show results similar to our prevalence of adolescent victimization and offending and childhood conventional ACEs. Additionally, past research has demonstrated the similarity between twins and singletons in antisocial behavior and risk factors for antisocial behavior [7].

## Conclusions

In this study, we have shown that the victim-offender overlap can be at least partly explained by early childhood characteristics and by the accumulation of multiple adverse childhood experiences, factors commonly used to identify “at risk” youth. Our results thus support the developmental axiom that past behavior and experience are predictive of future behavior and experience, and we extend this observation to show that it applies to the victim-offender overlap. Established delinquency-prevention programs have the opportunity to warn their clients about the risks of victimization and, perhaps, educate them on self-protective measures. Our study also showed that existing theories of the victim-offender overlap should be adapted to take a developmental perspective of risk beginning in childhood. Efforts to prevent adolescent victimization and offending may begin with a situational focus, such as programs to prevent retaliatory violence, but could improve their efficacy by recognizing that victimization and offending have their roots in childhood.

## Electronic supplementary material

ESM 1 (DOCX 85 kb)

## Footnotes

1 The *effect* of cumulative adversities that we discuss and measure differs from the *process* of cumulative disadvantage described in life-course theory [105], which describes how, through the process of labeling, young offenders experience subsequent life failures, leading to adult offending.

2 Internet and mobile phone harassment are criminal offenses under UK law. Details can be found here: http://www.cps.gov.uk/legal/a_to_c/communications_sent_via_social_media/ and here: http://www.cps.gov.uk/legal/s_to_u/stalking_and_harassment/#a05a

3 These latent factors are often abbreviated as A (additive genetic effect), C (shared environmental effect), and E (unique environmental effect), and the model is often called an ACE model. We avoid using the abbreviated terms in this study as they match the CDC’s abbreviation for adverse childhood experiences (ACEs).

4 For a complete review of twin models, modeling assumptions, and potential sources of bias in the estimated effects, we refer readers to Neale and Maes [81].

5 The percentages represent the standardized variances, or the proportion of variation explained by each given factor as calculated by ${V}_x^{\ast }={V}_x/{V}_p$, where *V*_*p*_ is the sum of all the unstandardized variance components and *V*_*x*_ is the unstandardized variance of a given factor.

6 The correlations were calculated using unstandardized forms of the variance components found in Table 1. The unstandardized variance components, standardized variance components, and the formulae for calculating the correlations between the factors can be found in Table S.5.

7 A common finding in behavioral genetic research is that genetics explain a greater amount of variance in a characteristic as subjects’ age. This, however, is not the case with antisocial behavior. Instead, the influence of both genetic and shared environmental factors tends to decline with age while the influence of unique environmental effects increases [102].
