# Supplementary material for: The Developmental Nature of the Victim-Offender Overlap
Source: J Dev Life Course Criminol. 2017 Oct 9;4(1):24–49. doi: 10.1007/s40865-017-0068-3 (PMC5865449; doi:10.1007/s40865-017-0068-3)
Supplement: Supplementary file 1 — (DOCX 85 kb) [file 40865_2017_68_MOESM1_ESM.docx]

**Online Supporting Information**

| **Table S.1. Sample and variable descriptions. (continued)** | | | | | | |
| --- | --- | --- | --- | --- | --- | --- |
| **Risk factor** | **Age** | **Source** | **Frequency (%)/**  **Mean (S.D.)** | **Description** | **N** | |
| **Analytic sample characteristics** | | |  |  |  | |
| Sex |  |  |  | Sex at birth | 2232 | |
| Female |  |  | 1140 (51.1%) |  |  | |
| Male |  |  | 1092 (48.9%) |  |  | |
| Zygosity |  | Mother-report |  | Zygosity was determined using a standard zygosity questionnaire that has been shown to have 95% accuracy (Price et al. 2000). Ambiguous cases were zygosity-typed using DNA. | 2232 | |
| Monozygotic |  |  | 1132 (54.5%) |  |  | |
| Dizygotic |  |  | 917 (45.5%) |  |  | |
| **Adolescent victimization and offending** | | | |  |  | |
| Victimization variety (up to 10) | 18 | Participant-report | 1.58 (2.54) | Additive variety scale of affirmative responses to 20 questions on victimization experiences between ages 13 and 18 years, tapping into 4 types of victimization: property crime victimization, physical assault, sexual assault (forced sex or touching by a known adult, forced sex or touching by a stranger, forced sexual behavior by a peer or sibling, completed or attempted forced intercourse), and internet/mobile harassment. Witnessing violence was not counted as part of this measure. Scores greater than 10 were re-coded to 10; 1% of Study members had scores greater than 10 (For additional details see Fisher et al. 2015). | 2059 | |
| Assault without weapon victim |  | 398 | 398 (19.3%) |  | 2065 | |
| Theft victim |  | 380 | 380 (18.4%) |  | 2066 | |
| Peer attack victim |  | 288 | 288 (13.9%) |  | 2063 | |
| Internet harassment victim |  | 285 | 285 (13.8%) |  | 2064 | |
| Vandalism victim |  | 276 | 276 (13.4%) |  | 2063 | |
| Mobile harassment victim |  | 225 | 225 (10.9%) |  | 2066 | |
| Assault with weapon victim |  | 206 | 206 (10.0%) |  | 2065 | |
| Other attack victim |  | 197 | 197 (9.5%) |  | 2066 | |
| Gang attack victim |  | 189 | 189 (9.1%) |  | 2066 | |
| Robbery victim |  | 161 | 161 (7.8%) |  | 2066 | |
| Slapped by partner |  | 117 | 117 (5.7%) |  | 2066 | |
| Genital attack victim |  | 115 | 115 (5.6%) |  | 2063 | |
| Internet sexual harassment victim |  | 114 | 114 (5.5%) |  | 2058 | |
| Sexual assault victim |  | 106 | 106 (5.2%) |  | 2066 | |
| Abuse by adult victm |  | 97 | 97 (4.7%) |  | 2059 | |
| Sexual coercion by peer victim |  | 45 | 45 (2.2%) |  | 2065 | |
| Assault because of skin color, religion, family origin, disability, or sexual orientation victim |  | 42 | 42 (2.0%) |  | 2065 | |
| Kidnap/attempted kidnap victim |  | 34 | 34 (1.6%) |  | 2059 | |
| Sexual assault by stranger victim |  | 32 | 32 (1.6%) |  | 2065 | |
| Sexual assault by known adult victim |  | 23 | 23 (1.1%) |  | 2065 | |
| Offending variety (up to 10) | 18 | Participant-report | 1.74 (2.67) | Additive variety scale of affirmative responses to 33 yes or no questions on offending behaviors between ages 17 and 18 years. 13 items tapped into violent offenses, 20 items tapped into non-violent offenses. Scores greater than 10 were re-coded to 10; 4% of Study members had scores greater than 10. | 2053 | |
| Stolen |  |  | 375 (18.3%) |  | 2052 | |
| Shoplifted |  |  | 374 (18.2%) |  | 2053 | |
| Hit during argument |  |  | 358 (17.4%) |  | 2053 | |
| Driven car or motorcycle without driver’s license |  |  | 278 (13.5%) |  | 2053 | |
| Damaged/destroyed school, college, university, or work property |  |  | 275 (13.4%) |  | 2053 | |
| Bully/threaten brother or sister |  |  | 248 (12.1%) |  | 2051 | |
| Painted or written graffiti |  |  | 190 (9.3%) |  | 2053 | |
| Damaged others’ property |  |  | 182 (8.9%) |  | 2053 | |
| Start fights |  |  | 160 (7.8%) |  | 2053 | |
| Sold stolen goods |  |  | 159 (7.7%) |  | 2053 | |
| Broken into another type of building |  |  | 118 (5.7%) |  | 2053 | |
| Taken part in happyslapping |  |  | 113 (5.5%) |  | 2053 | |
| Been in gang fight |  |  | 98 (4.8%) |  | 2052 | |
| Carried hidden weapon |  |  | 92 (4.5%) |  | 2053 | |
| Damaged parked car |  |  | 90 (4.4%) |  | 2053 | |
| Hurt someone to be nasty |  |  | 85 (4.1%) |  | 2053 | |
| Sold cannabis |  |  | 78 (3.8%) |  | 2052 | |
| Bully/threaten anyone |  |  | 76 (3.7%) |  | 2053 | |
| Hurt someone for fun |  |  | 69 (3.4%) |  | 2053 | |
| Driven a car dangerously or under the influence of alcohol or drugs |  |  | 68 (3.3%) |  | 2053 | |
| Hurt an animal on purpose |  |  | 50 (2.4%) |  | 2053 | |
| Used weapon on someone |  |  | 47 (2.3%) |  | 2051 | |
| Interfered with work of police |  |  | 40 (1.9%) |  | 2052 | |
| Taken part in riot |  |  | 38 (1.9%) |  | 2053 | |
| Used someone’s bank card without permission |  |  | 30 (1.5%) |  | 2053 | |
| Threatened someone to get money or items |  |  | 28 (1.4%) |  | 2053 | |
| Stolen car or motorcycle |  |  | 25 (1.2%) |  | 2052 | |
| Broken into someone’s house or flat |  |  | 24 (1.2%) |  | 2052 | |
| Broken into someone’s car |  |  | 23 (1.1%) |  | 2053 | |
| Hit parents or step-parents |  |  | 20 (1.0%) |  | 2053 | |
| Sold hard drugs |  |  | 16 (0.8%) |  | 2052 | |
| Committed identity fraud |  |  | 16 (0.8%) |  | 2053 | |
| Cloned someone’s bank card |  |  | 5 (0.2%) |  | 2053 | |
| Official record | 10-18 | UK Police National Computer (PNC) | 210 (10.2%) | UK Police National Computer (PNC) records were searched for E-risk cohort members by the UK Ministry of Justice (MoJ). The PNC includes complete history of cautions and convictions after age 10 years for individuals cautioned or convicted in the UK from the year 2000 until February 2016. Because E-risk cohort members were ca. aged 6 years when the PNC records began, the PNC comprises their complete offending histories, covering approximately a 12-year observation period from age 10 years forward. Of the cohort, 2060 twins gave informed consent for the search at the age-18 interview (99.3% of those taking part at age 18; 92.3% of the original cohort). Attrition analysis showed that prevalence of lifetime diagnosed childhood conduct disorder did not differ significantly between cohort members whose records were searched versus were not searched (15.8% versus 15.8%). | 2060 | |
| **Personal risk factors** | | |  |  |  | |
| Low self-control z-score | 5-10 | Mother-, Teacher-, Interviewer-, and self-report | 0 (1.00) | Children’s self-control during their first decade of life was measured using a multi-occasion/multi-informant strategy, following Moffitt et al. (2011). Briefly, a self-control factor was estimated via nine measures, including observational ratings of children’s lack of control (age 5 years), parent and teacher reports of poor impulse control (ages 5, 7 and 10 years), self-reports of inattentive and impulsive behavior (age 7 years), and interviewer judgements of the personality trait of conscientiousness (age 10 years). | 2232 | |
| Cognitive ability | 12 | Test | 98.64 (15.60) | Test score with a full-sample mean of 100 and full-sample standard deviation of 15. Tested by using a short form of the Wechsler Intelligence Scale for Children, Fourth Edition (2003). | 2131 | |
| Early puberty | 12 | Parent-report | 2.64 (1.11) | Rank score of participant’s physical development according to the highest ranked of standard photographs depicting 5 stages of puberty on the Tanner scale(Marshall and Tanner 1969, 1970). | 2052 | |
| Conduct disorder diagnosis | 5-12 | Mother- and Teacher-report | 349 (15.8%) | Dichotomous indicator of conduct disorder. Conduct disorder was measured according to the symptom criteria of the Diagnostic and Statistical Manual of Mental Disorders (DSM-IV; American Psychiatric Association 1994), which identify adolescents displaying a persistent pattern of behavior that violates the rights of others, including physical harm. A diagnosis of conduct disorder (using a 12-month reporting period for symptoms) was made at each of four ages: ages 5, 7, 10, and 12. Study participants were recorded as having conduct disorder if five or more conduct disorder symptoms were reported at any of the four waves. (For additional details see Kim-Cohen et al. 2005) | 2214 | |
| Childhood delinquency | 12 | Participant-report | 1.92 (1.97) | Additive variety scale of affirmative responses to 24 questions on delinquent behavior. All items were speciﬁcally selected to map onto the DSM–IV criteria for conduct disorder (American Psychiatric Association 1994). Behaviors included: running away overnight (runaway), purposefully destroying or damaging property (vandalism), breaking into a building to steal something (breaking & entering), theft, taking something from a store without paying for it (shoplifting), bullying/threatening, theft from a vehicle, physically assaulting, and hurting animals. | 2120 | |
| Childhood substance use | 12 | Participant-report | 0.85 (1.17) | Scale of frequency of use of drinking alcohol, smoking cigarettes, hash or cannabis use, pharmaceutical use, glue/gas sniffing. Participants responded with No, Yes – only once or twice, or Yes – more than twice. (Mayer and Filstead 1979; Currie et al. 2004) | 2117 | |
| **Total count of all 19 ACEs** |  |  | 3.15 (2.63) |  | 2232 | |
| **Conventional ACEs (up to 9 ACEs)** |  |  | 1.49 (1.55) |  | 2232 | |
| Physical abuse | Birth-12 | Life dossier | 114 (5.1%) | Dichotomous indicator of severe maltreatment. The study developed a cumulative profile for each child across ages 5, 7, 10, and 12. The profile comprised the caregiver reports of maltreatment, recorded debriefings with interviewers who had coded any indication of maltreatment at any of the successive home visits, recorded narratives of the successive caregiver interviews, and information from clinicians whenever the study team made a child-protection referral. The profiles were reviewed at the end of the age 12 phase by two clinical psychologists. Initial inter-rater agreement between the coders was 90% of cases for whom maltreatment was identified (100% for cases of sexual abuse), and discrepantly coded cases were resolved by consensus review (For additional details see Danese et al. 2016). | 2232 | |
| Sexual abuse | Birth-12 | Life dossier | 16 (0.7%) |  | 2232 | |
| Emotional abuse or neglect | Birth-12 | Life dossier | 68 (3.1%) |  | 2232 | |
| Physical neglect | Birth-12 | Life dossier | 44 (2.0%) | Dichotomous indicator of severe physical neglect defined as any sign that the caretaker was not providing a safe, sanitary, or healthy environment for the child. This included the child not having proper clothing or food, as well as grossly unsanitary home environments. Initial inter-rater agreement between the coders was 85% of cases for whom maltreatment was identified, and discrepantly coded cases were resolved by consensus review (For additional details see Danese et al. 2016).. | 2232 | |
| Domestic violence exposure | Birth-10 | Mother-report | 374 (16.8%) | Dichotomous indicator of exposure to severe domestic violence as reported by mothers. Mothers reported about perpetration of and victimization involving 12 forms of physical violence (e.g., slapping, hitting, kicking, and strangling) from the Conflict Tactics Scale (Straus 1990), when the children were 5, 7, and 10 years of age). Reports of either perpetration or victimization constituted evidence of physical domestic violence (For additional details see Danese et al. 2016). | 2232 | |
| Parental antisocial behavior | 5 | Mother-report | 560 (25.2%) | Dichotomous indicator of approximately the top quarter of the variety of antisocial behaviors of parents. Father’s and mother’s history of antisocial behavior was reported by the mothers when the children were 5 years old. Mothers were interviewed using the Young Adult Behavior Checklist (Achenbach 1997), which was modified to obtain lifetime data. Full details of father’s and mother’s history of antisocial behavior within the E-Risk Study are reported elsewhere (Jaffee et al. 2003). A study of mother–father agreement about men’s antisocial behavior in this sample showed that women provided reliable information about the behavior of their children’s father (Caspi et al. 2001). | 2226 | |
| Family history of substance abuse | 12 | Mother-report | 478 (22.3%) | Dichotomous indicator of approximately the top quarter of the proportion of family members with a history of substance use for any of the child’s biological mother, father, maternal grandparents, or maternal aunts and uncles. Family history algorithms follow those outlined by Milne et al. (2008). | 2140 | |
| Family history of mental health disorders | 12 | Mother-report | 658 (31.0%) | Dichotomous indicator of a family history of a report of hospitalization for psychiatric disorder or attempted or completed suicide for any of the child’s biological mother, father, maternal grandparents, or maternal aunts and uncles (For additional details see Belsky et al. 2012). | 2120 | |
| Parental separation or divorce | Birth-10 | Mother-report | 1024 (48.0%) | Dichotomous indicator of biological parent separation or divorce as assessed at each interview, up to 10 years of age, by questions on whether the biological parents were living in the same household. | 2132 | |
| **Expanded ACEs (up to 10 ACEs)** |  |  | 1.66 (1.61) |  | 2232 | |
| Bullying victim | 5-12 | Mother-report  Participant-report | 197 (8.9%) | Dichotomous indicator of severe bullying, operationally defined as evidence of (a) repeated harmful actions, (b) between children, and (c) where there is a power differential between the bully and the victim. Full details about bullying are reported in Shakoor et al. (2012). | 2214 | |
| Lived in foster care | 5-12 | Life dossier | 24 (1.1%) | Dichotomous indicator of whether the participant had lived in foster care during their lifetime. | 2232 | |
| Low socioeconomic status | 5 | Mother-report | 442 (19.8%) | Dichotomous indicator of approximately the top quarter of socioeconomic disadvantage, defined as follows: (a) head of household had no educational qualifications; (b) head of household was employed in an unskilled occupation or was not in the labor force; (c) total household gross annual income was less than £10,000; (d) family was receiving at least one government benefit, excluding disability benefit; (e) family housing was government subsidized; and (f) family had no access to a vehicle (For additional details see Kim-Cohen et al. 2004). | 2232 | |
| Peer substance abuse | 12 | Participant-report | 421 (19.9%) | Dichotomous indicator of approximately the top quarter of scores on a scale of five items. The items capture, for each twin separately, the number of peers who drink alcohol, smoke cigarettes, use hash or cannabis, use pharmaceuticals, sniff glue/gas. Participants responded with none, some, most, or all. | 2118 | |
| Low parental monitoring (mother) | 12 | Mother-report | 396 (18.5%) | Dichotomous indicator of approximately the top quarter of scores on a scale of ten items from the Monitoring and Supervision Questionnaire (Stattin and Kerr 2000). Mothers were asked about their monitoring and knowledge during the last 6 months. The items capture, for each twin separately, whether the child needed permission to leave home or before deciding what to do on the weekend, and whether they had to report on where and who they go out with. Mothers also reported on whether they knew the friends their child hangs out with, where they go in their spare time, how they spend their money, what type of homework or tests and projects they have, and how their child performs in different subjects. Answers were recorded as no, never (0), sometimes (1) and yes, always (2) (For additional details see Wertz et al. 2016). | 2141 | |
| Low parental monitoring (participant) | 12 | Participant-report | 475 (22.4%) | Dichotomous indicator of approximately the top quarter of scores using the same items used with mother-rated parental monitoring but worded slightly differently (e.g. Do your parents know…) (For additional details see Wertz et al. 2016) | 2120 | |
| Participant-perceived unsafe neighborhood | 12 | Participant-report | 260 (12.3%) | Dichotomous indicator of whether the participant felt unsafe in their neighborhood. Question: You feel unsafe in your neighborhood. Participants responded with true or false. | 2108 | |
| High neighbor victimization | 13-14 | Neighbor survey | 542 (25.2%) | Dichotomous indicator of approximately the top quarter of scores on a scale of three items tapping into neighbor victimization experiences. Neighbors in the same postal code as the Study member were surveyed on whether they had been a victim of 3 different types of crime (home break-in, theft from outdoor home property, violence experienced by respondent or family member in neighborhood). Neighbors were able to respond with no, yes, once, and yes, more than once, for each of the three types of victimization. Complete details on the survey methodology can be found in. (Complete details on the survey methodology can be found in Odgers et al. 2009). | 2148 | |
| Neighborhood rated unsafe | 14-16 | Systematic social observation (SSO) | 410 (20.5%) | Dichotomous indicator of approximately the top quarter of the average scores on two items assessing whether the neighborhood appears unsafe. As part of the Community Strengths project, raters from the research team used Google Street View images of each Study member’s neighborhood to respond to two questions on the neighborhood’s appearance: whether the raters felt that the neighborhood ‘a safe place to live?’ and ‘somewhere they would feel safe walking at night?’. Raters were able to provide scores ranging from definitely safe (1) to definitely unsafe (5). (Complete details on the SSO methodology can be found in Odgers, Bates, et al. 2009) | 2004 | |
| High-crime neighborhood | 15-17 | Police data | 534 (25.0%) | Dichotomous indicator where 1 equals the top quarter of police-recorded crime and 0 equals the bottom three-quarters. *Local area crime* was measured by mapping a 1 mile radius around each E-Risk Study family’s home and tallying the total number of crimes that occurred in the area each month. Street-level crime data, including information on the type of crime, date of occurrence, and approximate location, were accessed online as part of an open data sharing effort about crime and policing in England and Wales (https://data.police.uk/). An Application Program Interface (API) was used to extract street-level crime data for each of the geospatial coordinates marking the family’s home. For a full description see: <https://data.police.uk/about/#location-anonymisation> The monthly average of the total number of crimes for the area surrounding each study member family’s home was computed for 2011, the first year for which full street-level data was available (and also in the year prior to the start of the age 18 assessments ). | 2134 | |
| **Summary scale of all risk factors** |  |  | 1.99 (1.39) | Measure of all risk factors (personal risk factors, conventional ACEs, expanded ACEs). For the summary scale Study members were counted as having the risk factor if they were: in the top quartile of low self-control, early puberty, childhood self-reported delinquency, and childhood substance use; in the bottom quartile of cognitive ability; met the diagnostic criteria for conduct disorder. |  | |
| Note: S.D. – standard deviation | | | | | | |

**References for Table S.1.**

Achenbach, Thomas M. 1997. *Young Adult Self Report*. Burlington, VT: University of Vermont, Department of Psychiatry.

American Psychiatric Association. 1994. *Diagnostic and Statistical Manual of Mental Disorders*. 4th ed. Washington, DC: APA.

Belsky, Daniel W., Avshalom Caspi, Louise Arseneault, Wiebke Bleidorn, Peter Fonagy, Marianne Goodman, Renate Houts, and Terrie E. Moffitt. 2012. “Etiological Features of Borderline Personality Related Characteristics in a Birth Cohort of 12-Year-Old Children.” *Development and Psychopathology* 24 (01): 251–265. doi:10.1017/S0954579411000812.

Caspi, Avshalom, Alan Taylor, Matt Smart, Jan Jackson, Steve Tagami, and Terrie E. Moffitt. 2001. “Can Women Provide Reliable Information about Their Children’s Fathers? Cross-Informant Agreement about Men’s Lifetime Antisocial Behaviour.” *The Journal of Child Psychology and Psychiatry and Allied Disciplines* 42 (7): 915–20. doi:10.1017/S0021963001007636.

Currie, Candace, Chris Roberts, Antony Morgan, Rebecca Smith, Wolfgang Settertobulte, Oddrun Samdal, and Vivian Barnekow Rasmussen. 2004. “Young People’s Health in Context Health Behaviour in School-Aged Children (HBSC) Study: International Report from the 2001/2002 Survey.” Health Policy for Children and Adolescents, No. 4. World Health Organization.

Danese, Andrea, Terrie E. Moffitt, Louise Arseneault, Ben A. Bleiberg, Perry B. Dinardo, Stephanie B. Gandelman, Renate Houts, et al. 2016. “The Origins of Cognitive Deficits in Victimized Children: Implications for Neuroscientists and Clinicians.” *American Journal of Psychiatry* 174 (4): 349–61. doi:10.1176/appi.ajp.2016.16030333.

Fisher, Helen L., Avshalom Caspi, Terrie E. Moffitt, Jasmin Wertz, Rebecca Gray, Joanne Newbury, Antony Ambler, et al. 2015. “Measuring Adolescents’ Exposure to Victimization: The Environmental Risk (E-Risk) Longitudinal Twin Study.” *Development and Psychopathology* 27 (November): 1399–1416. doi:10.1017/S0954579415000838.

Jaffee, Sara R., Terrie E. Moffitt, Avshalom Caspi, and Alan Taylor. 2003. “Life with (or without) Father: The Benefits of Living with Two Biological Parents Depend on the Father’s Antisocial Behavior.” *Child Development* 74 (1): 109–26. doi:10.1111/1467-8624.t01-1-00524.

Kim-Cohen, Julia, Louise Arseneault, Avshalom Caspi, Mónica Polo Tomás, Alan Taylor, and Terrie E. Moffitt. 2005. “Validity of DSM-IV Conduct Disorder in 4½–5-Year-Old Children: A Longitudinal Epidemiological Study.” *American Journal of Psychiatry* 162 (6): 1108–17. doi:10.1176/appi.ajp.162.6.1108.

Kim-Cohen, Julia, Terrie E. Moffitt, Avshalom Caspi, and Alan Taylor. 2004. “Genetic and Environmental Processes in Young Children’s Resilience and Vulnerability to Socioeconomic Deprivation.” *Child Development* 75 (3): 651–68. doi:10.1111/j.1467-8624.2004.00699.x.

Marshall, W. A., and J. M. Tanner. 1969. “Variations in Pattern of Pubertal Changes in Girls.” *Archives of Disease in Childhood* 44 (235): 291–303.

———. 1970. “Variations in the Pattern of Pubertal Changes in Boys.” *Archives of Disease in Childhood* 45 (239): 13–23. doi:10.1136/adc.45.239.13.

Mayer, J, and W J Filstead. 1979. “The Adolescent Alcohol Involvement Scale. An Instrument for Measuring Adolescents’ Use and Misuse of Alcohol.” *Journal of Studies on Alcohol* 40 (3): 291–300. doi:10.15288/jsa.1979.40.291.

Milne, B. J., T. E. Moffitt, R. Crump, R. Poulton, M. Rutter, M. R. Sears, A. Taylor, and A. Caspi. 2008. “How Should We Construct Psychiatric Family History Scores? A Comparison of Alternative Approaches from the Dunedin Family Health History Study.” *Psychological Medicine* 38 (12): 1793–1802. doi:10.1017/S0033291708003115.

Moffitt, Terrie E., Louise Arseneault, Daniel Belsky, Nigel Dickson, Robert J. Hancox, HonaLee Harrington, Renate Houts, et al. 2011. “A Gradient of Childhood Self-Control Predicts Health, Wealth, and Public Safety.” *Proceedings of the National Academy of Sciences* 108 (7): 2693–98. doi:10.1073/pnas.1010076108.

Odgers, Candice L., Christopher J. Bates, Avshalom Caspi, Robert J. Sampson, and Terrie E. Moffitt. 2009. “Systematic Social Observation Inventory–tally of Observations in Urban Regions (SSO I-Tour).” *Measurement* 12: 21–34.

Odgers, Candice L., Terrie E. Moffitt, Laura M. Tach, Robert J. Sampson, Alan Taylor, Charlotte L. Matthews, and Avshalom Caspi. 2009. “The Protective Effects of Neighborhood Collective Efficacy on British Children Growing up in Deprivation: A Developmental Analysis.” *Developmental Psychology* 45 (4): 942–57. doi:10.1037/a0016162.

Price, Thomas S., Bernard Freeman, Ian Craig, Stephen A. Petrill, Lorna Ebersole, and Robert Plomin. 2000. “Infant Zygosity Can Be Assigned by Parental Report Questionnaire Data.” *Twin Research and Human Genetics* 3 (3): 129–33. doi:10.1375/twin.3.3.129.

Shakoor, Sania, Sara R. Jaffee, Lucy Bowes, Isabelle Ouellet-Morin, Penelope Andreou, Francesca Happé, Terrie E. Moffitt, and Louise Arseneault. 2012. “A Prospective Longitudinal Study of Children’s Theory of Mind and Adolescent Involvement in Bullying.” *Journal of Child Psychology and Psychiatry* 53 (3): 254–61. doi:10.1111/j.1469-7610.2011.02488.x.

Stattin, Häkan, and Margaret Kerr. 2000. “Parental Monitoring: A Reinterpretation.” *Child Development* 71 (4): 1072–85. doi:10.1111/1467-8624.00210.

Straus, Murray A. 1990. “New Scoring Methods for Violence and New Norms for the Conflict Tactics Scales.” In *Physical Violence in American Families: Risk Factors and Adaptations to Violence in 8,145 Families*, edited by Murray A. Straus and Richard J. Gelles, 535–60. New Brunswick, NJ: Transaction Books.

Wechsler, David. 2003. *The Wechsler Intelligence Scale for Children.* 4th ed. London: Pearson.

Wertz, Jasmin, Kate Nottingham, Jessica Agnew-Blais, Timothy Matthews, Carmine M. Pariante, Terrie E. Moffitt, and Louise Arseneault. 2016. “Parental Monitoring and Knowledge: Testing Bidirectional Associations with Youths’ Antisocial Behavior.” *Development and Psychopathology* 28 (3): 623–38. doi:10.1017/S0954579416000213.

| **Table S.2 Types of victimization experienced by Study members. Single-type victimization and multiple-type victimization were almost equally likely. Table 4 model results robust to victim-offender categories based on single-type victimization.** | | | | | | |
| --- | --- | --- | --- | --- | --- | --- |
|  | **Property crime** | **Physical assault** | **Sexual assault** | **Internet/mobile harassment** | ***n*** | % |
| **No victimization** |  |  |  |  | 1112 | 54.01% |
|  |  |  |  |  |  |  |
| **Single victimization** |  |  |  |  | 508 | 24.67% |
|  | X |  |  |  | 218 | 10.59% |
|  |  | X |  |  | 147 | 7.14% |
|  |  |  |  | X | 125 | 6.07% |
|  |  |  | X |  | 18 | 0.87% |
|  |  |  |  |  |  |  |
| **Multiple victimizations** |  |  |  |  | 439 | 21.32% |
|  | X | X |  |  | 98 | 4.76% |
|  | X |  |  | X | 79 | 3.84% |
|  | X | X |  | X | 79 | 3.84% |
|  |  | X |  | X | 54 | 2.62% |
|  | X | X | X | X | 46 | 2.23% |
|  | X | X | X |  | 18 | 0.87% |
|  |  |  | X | X | 15 | 0.73% |
|  |  | X | X | X | 15 | 0.73% |
|  | X |  | X | X | 12 | 0.58% |
|  |  | X | X |  | 12 | 0.58% |
|  | X |  | X |  | 11 | 0.53% |
| **Total** |  |  |  |  | 2059 | 100.00% |

|  | | | | | | | | | | | | | | | | | | | | |
| --- | --- | --- | --- | --- | --- | --- | --- | --- | --- | --- | --- | --- | --- | --- | --- | --- | --- | --- | --- | --- |
| **Table S.3. Correlation matrix of ACEs, 9 original ACEs and 10 expanded ACEs.** | | | | | | | | | | | | | | | | | | | | |
|  | | 1 | 2 | 3 | 4 | 5 | 6 | 7 | 8 | 9 | 10 | 11 | 12 | 13 | 14 | 15 | 16 | 17 | 18 |  |
| 1- Physical abuse | | 1 |  |  |  |  |  |  |  |  |  |  |  |  |  |  |  |  |  |  |
| 2- Sexual abuse | | 0.18 | 1 |  |  |  |  |  |  |  |  |  |  |  |  |  |  |  |  |  |
| 3- Emotional abuse or neglect | | 0.84 | 0.38 | 1 |  |  |  |  |  |  |  |  |  |  |  |  |  |  |  |  |
| 4- Physical neglect | | 0.67 | 0.35 | 0.66 | 1 |  |  |  |  |  |  |  |  |  |  |  |  |  |  |  |
| 5- Domestic violence exposure | | 0.44 | 0.24 | 0.48 | 0.46 | 1 |  |  |  |  |  |  |  |  |  |  |  |  |  |  |
| 6- Parental antisocial behavior | | 0.49 | 0.19 | 0.48 | 0.53 | 0.58 | 1 |  |  |  |  |  |  |  |  |  |  |  |  |  |
| 7- Family history of substance abuse | | 0.37 | 0.03 | 0.26 | 0.37 | 0.37 | 0.51 | 1 |  |  |  |  |  |  |  |  |  |  |  |  |
| 8- Family history of mental health disorders | | 0.27 | 0.00 | 0.32 | 0.33 | 0.36 | 0.33 | 0.53 | 1 |  |  |  |  |  |  |  |  |  |  |  |
| 9- Parental separation or divorce | | 0.46 | 0.07 | 0.47 | 0.42 | 0.41 | 0.65 | 0.34 | 0.23 | 1 |  |  |  |  |  |  |  |  |  |  |
| 10- Bullying victim | | 0.32 | 0.32 | 0.27 | 0.40 | 0.15 | 0.20 | 0.15 | 0.26 | 0.20 | 1 |  |  |  |  |  |  |  |  |  |
| 11- Lived in foster care | | 0.71 | 0.15 | 0.55 | 0.68 | 0.28 | 0.48 | 0.53 | 0.19 | 0.59 | 0.30 | 1 |  |  |  |  |  |  |  |  |
| 12- Low socioeconomic status | | 0.35 | 0.30 | 0.42 | 0.56 | 0.36 | 0.55 | 0.20 | 0.17 | 0.53 | 0.21 | 0.57 | 1 |  |  |  |  |  |  |  |
| 13- Peer substance abuse | | 0.17 | 0.25 | 0.18 | 0.26 | 0.11 | 0.23 | 0.19 | 0.18 | 0.18 | 0.18 | 0.22 | 0.17 | 1 |  |  |  |  |  |  |
| 14- Low parental monitoring (mother) | | 0.33 | 0.08 | 0.28 | 0.37 | 0.13 | 0.28 | 0.11 | 0.11 | 0.16 | 0.20 | 0.32 | 0.25 | 0.29 | 1 |  |  |  |  |  |
| 15- Low parental monitoring (participant) | | 0.20 | 0.16 | 0.28 | 0.02 | 0.20 | 0.23 | 0.13 | 0.14 | 0.19 | 0.15 | -0.10 | 0.23 | 0.34 | 0.35 | 1 |  |  |  |  |
| 16- Participant-perceived unsafe neighborhood | | 0.20 | 0.10 | 0.17 | 0.25 | 0.14 | 0.16 | 0.13 | 0.13 | 0.21 | 0.42 | 0.30 | 0.22 | 0.18 | 0.04 | 0.13 | 1 |  |  |  |
| 17- High neighbor victimization | | 0.11 | 0.13 | 0.13 | 0.16 | 0.11 | 0.24 | 0.14 | 0.12 | 0.27 | 0.08 | 0.21 | 0.40 | 0.20 | 0.12 | 0.18 | 0.26 | 1 |  |  |
| 18- Neighborhood appears unsafe | | 0.14 | 0.18 | 0.20 | 0.34 | 0.16 | 0.28 | 0.17 | 0.13 | 0.14 | 0.15 | 0.42 | 0.50 | 0.21 | 0.22 | 0.07 | 0.30 | 0.42 | 1 |  |
| 19- High-crime neighborhood | | 0.13 | 0.00 | 0.22 | 0.11 | 0.17 | 0.18 | 0.02 | 0.05 | 0.18 | 0.06 | 0.02 | 0.32 | -0.02 | -0.02 | 0.00 | 0.29 | 0.34 | 0.36 |  |
| Notes: Readers may be concerned about double-counting of ACEs items. However, as the table indicates, estimates of parental monitoring vary depending on the reporting source (Stattin and Kerr 2000; Wertz et al. 2016). Likewise, the four neighborhood variables were derived from four different sources: self-reports, neighbor surveys, systematic social observation via Google Street View, and police administrative data, respectively, and are only modestly correlated.  Pairwise tetrachoric correlations (rho). | | | | | | | | | | | | | | | | | | | |  |

| **Table S.4. Model fit statistics for bivariate twin model indicating that the best fitting model included genetic and environmental factors.** | | | | | | | |
| --- | --- | --- | --- | --- | --- | --- | --- |
| **Model** | **Number of parameters** | **-2 log likelihood (2ll)** | **Degrees of freedom (df)** | **AIC** | **∆ 2ll** | **∆ df** | **Likelihood ratio test p-value** |
| ACE | 11 | 18692.79 | 4101 | 10490.79 |  |  |  |
| AE | 8 | 18703.18 | 4104 | 10495.18 | 10.39 | 3 | 0.016 |
| CE | 8 | 18718.35 | 4104 | 10510.35 | 25.56 | 3 | < 0.001 |
| E | 5 | 19138.05 | 4107 | 10924.05 | 445.26 | 6 | < 0.001 |
| Notes: A refers to additive genetic effect, C refers to shared environmental effect, E refers to unique environmental effect. | | | | | | | |

| **Table S.5. Variance components from bivariate twin model of victimization and offending at age 18 years, 95% confidence interval in parentheses.** | | | |
| --- | --- | --- | --- |
|  | **Additive genetic (A)** | **Shared environment (C)** | **Unique environment (E)** |
| **Unstandardized** |  |  |  |
| Victimization | 2.01  (0.78 – 3.23) | 1.23  (0.20 – 2.31) | 3.23  (3.23 – 3.62) |
| Offending | 2.35  (1.08 – 3.69) | 1.55  (0.39 – 2.67) | 3.22  (2.88 – 3.63) |
| Victimization-offending | 0.61  (0 – 1.59) | 1.19  (0.33 – 2.01) | 1.07  (0.80 – 1.36) |
| **Standardized** |  |  |  |
| Victimization | .32  (0.13 – 0.50) | .18  (0.02 – 0.34) | .50  (0.45 – 0.57) |
| Offending | .33  (0.15 – 0.51) | .22  (0.06 – 0.37) | .45  (0.40 – 0.51) |
| Victimization-offending | .22  (0 – 0.56) | .40  (0.10 – 0.68) | .37  (0.28 – 0.48) |
| Note: The cross-twin, cross-trait correlations were constrained to the values for Offending Twin A-Victimization Twin B, as reported in Table 2. | | | |

| **Table S.6. Estimated correlations between victimization and offending, by zygosity and sex, in twin pairs, 95% confidence interval in parentheses.** | | | |
| --- | --- | --- | --- |
|  | Victimization Twin B | | Offending Twin B |
| **Monozygotic Males** |  |  |  |
|  |  | |  |
| Victimization Twin A | 0.49  (0.39 – 0.57) | |  |
|  |  |  |  |
| Offending Twin B | 0.25  (0.17 – 0.33) | | 0.52  (0.43 – 0.60) |
|  |  |  |  |
| **Dizygotic Males** |  |  |  |
|  |  |  |  |
| Victimization Twin A | 0.26  (0.12 – 0.38) | |  |
| Offending Twin B | 0.20  (0.10 – 0.30) | | 0.30  (0.18 – 0.41) |
| **Monozygotic Females** |  |  |  |
|  |  | |  |
| Victimization Twin A | 0.51  (0.43 – 0.59) | |  |
|  |  |  |  |
| Offending Twin B | 0.23  (0.15 – 0.30) | | 0.46  (0.38 – 0.54) |
|  |  |  |  |
| **Dizygotic Females** |  |  |  |
|  |  |  |  |
| Victimization Twin A | 0.43  (0.32 – 0.52) | |  |
| Offending Twin B | 0.20  (0.11 – 0.28) | | 0.39  (0.27 – 0.50) |
| Note: The correlation between victimization and offending in the full cohort was 0.42. The victimization-offending correlations did not significantly differ by sex. The correlation among monozygotic male twins was 0.44 (95% CI: 0.36 – 0.51). The correlation among dizygotic male twins was 0.45 (95% CI: 0.37-0.52). The correlation among monozygotic female twins was 0.31 (95% CI: 0.23 – 0.39). The correlation among dizygotic female twins was 0.44 (95% CI: 0.36-0.51). | | | |

| **Table S.7. Sensitivity analysis: Using official-record-based offender status to compare victim-offenders to three groups (individuals who are neither victims nor offenders, victims-only, and offenders-only).** | | | | | |
| --- | --- | --- | --- | --- | --- |
| **Risk factor** | **Victim-offender status** | | | | ***p* < .05 compared to victim-offender^a^** |
|  | **Neither**  **(N)  N=983** | **Victim only**  **(V) N=735** | **Offender only**  **(O)  N=55** | **Victim-offender N=122** |  |
| **Personal risk factors** |  |  |  |  |  |
| Low self-control z-score, mean (SD) | -0.27 (0.91) | 0.09 (0.97) | 0.74 (1.06) | 0.76 (1.00) | N, V |
| Cognitive ability, mean (SD) | 100.59 (15.25) | 99.36 (14.86) | 88.45 (14.11) | 88.58 (15.41) | N, V |
| Early puberty, mean (SD) | 2.59 (1.09) | 2.72 (1.12) | 2.67 (1.11) | 2.63 (1.11) | - |
| Conduct disorder diagnosis, N (%) | 76 (7.7%) | 125 (17.0%) | 24 (43.6%) | 61 (50.0%) | N, V |
| Childhood self-reported delinquency, mean (SD) | 1.43 (1.69) | 2.19 (1.93) | 3.49 (2.91) | 3.21 (2.36) | N, V |
| Childhood substance use, mean (SD) | 0.62 (0.94) | 1.01 (1.25) | 1.48 (1.56) | 1.58 (1.59) | N, V |
| **Total of all ACEs (up to 19 ACEs), mean (SD)** | 2.42 (2.17) | 3.56 (2.65) | 4.80 (2.79) | 5.57 (2.96) | N, V |
| **Summary scale of all risk factors, mean (SD)** | 1.71 (1.18) | 2.30 (1.34) | 3.24 (1.54) | 3.03 (1.56) | N, V |
| Notes: ^a^ Significant two-tailed tests of mean or proportion difference between the victim-offender group and each of the other groups are noted by the single letter abbreviation of the group for which the significant difference was found: N = neither victim nor offender, V = victim-only, and O = offender-only. | | | | | |

**Method for sensitivity analysis:** We created a dichotomous measure of official record, by counting any recorded caution or conviction as “1”; 210 (10%) of E-Risk study members had a police record. We restricted our analysis to participants included in the person-centered approach (those with complete information on all of the main variables who consented to a records search, *n* = 1895). We then created cross-tabuled self-reported victimization and police-recorded offending to create categories of victim-offender status (neither (52%), victim-only (39%), offender-only (3%), victim-offender (6%)). 31 study members with a police record had self-reported no offending.
